# *ITPKC* Single Nucleotide Polymorphism Associated with the Kawasaki Disease in a Taiwanese Population

**DOI:** 10.1371/journal.pone.0017370

**Published:** 2011-04-14

**Authors:** Ho-Chang Kuo, Kuender D. Yang, Suh-Hang Hank Juo, Chi-Di Liang, Wei-Chiao Chen, Yu-Shiuan Wang, Chih-Hung Lee, Edward Hsi, Hong-Ren Yu, Peng-Yeong Woon, I-Chun Lin, Chien-Fu Huang, Daw-Yang Hwang, Chiu-Ping Lee, Li-Yan Lin, Wei-Pin Chang, Wei-Chiao Chang

**Affiliations:** 1 Division of Allergy, Immunology and Rheumatology, Department of Pediatrics and Clinical Genomic & Proteomic Core laboratory, Chang Gung Memorial Hospital-Kaohsiung Medical Center, Taiwan.; 2 Division of Cardiology, Department of Pediatrics, Chang Gung Memorial Hospital-Kaohsiung Medical Center, Kaohsiung, Taiwan; 3 Graduate Institute of Clinical Medical Science, Chang Gung University College of Medicine, Kaohsiung, Taiwan; 4 Department of Medical Genetics, College of Medicine, Kaohsiung Medical University, Kaohsiung, Taiwan; 5 Center of Excellence for Environmental Medicine, Kaohsiung Medical University, Kaohsiung, Taiwan; 6 Cancer Center, Kaohsiung Medical University Hospital, Kaohsiung, Taiwan; 7 Department of Healthcare Management, Yuanpei University, HsinChu, Taiwan; 8 Graduate Institute of Medicine, College of Medicine, Kaohsiung Medical University, Kaohsiung, Taiwan; 9 Department of Molecular Biology and Human Genetics, Tzu Chi University, Hualien, Taiwan; 10 Division of Nephrology, Department of Medicine, Kaohsiung Medical University Hospital, Kaohsiung, Taiwan; 11 Department of Dermatology, Graduate Institute of Medicine, Kaohsiung Medical University, Kaohsiung, Taiwan; Istituto Dermopatico dell'Immacolata, Italy

## Abstract

Kawasaki disease (KD) is characterized by systemic vasculitis with unknown etiology. Previous studies from Japan indicated that a gene polymorphism of *ITPKC* (rs28493229) is responsible for susceptibility to KD. We collected DNA samples from 1,531 Taiwanese subjects (341 KD patients and 1,190 controls) for genotyping *ITPKC*. In this study, no significant association was noted for the *ITPKC* polymorphism (rs28493229) between the controls and KD patients, although the CC genotype was overrepresented. We further combined our data with previously published case/control KD studies in the Taiwanese population and performed a meta-analysis. A significant association between rs28493229 and KD was found (Odds Ratio:1.36, 95% Confidence Interval 1.12–1.66). Importantly, a significant association was obtained between rs28493229 and KD patients with aneurysm formation (*P* = 0.001, under the recessive model). Taken together, our results indicated that C-allele of *ITPKC* SNP rs28493229 is associated with the susceptibility and aneurysm formation in KD patients in a Taiwanese population.

## Introduction

Kawasaki disease (KD) is a multi-systemic vasculitis with unknown etiology which was first reported by Kawasaki et al. [1] from Japan in 1974 in the English language literature. It occurs worldwide and mainly affects children less than 5 years old especially in Asian countries, Japan, Korea and Taiwan with the incidence from 69 to 213 cases per 100000 for children <5 years of age [2], [3]. The clinical characteristics of KD include prolonged fever, bilateral non-purulent conjunctivitis, diffuse mucosal inflammation, polymorphous skin rashes, indurative angioedema of the hands and feet, and non-suppurative cervical lymphadenopathy [3], [4]. The most serious complication of KD is coronary artery lesions (CAL) including myocardial infarction, coronary artery dilatation, coronary fistula or coronary artery aneurysm [5], [6]. In developed countries, KD is the leading cause of acquired heart diseases in children.

While these clinical features of KD are recognized, their underlying immunopathogenic mechanisms are still unclear; in particular, the culprit for developing CAL in patients with KD remains as yet to be uncovered. KD is regarded as an immune disorder of host rather than an infectious disease. From our previous reports, monocytosis and T helper 2 (Th2) immune response were associated with CAL formation [6], [7], and intravenous immunoglobulin (IVIG) treatment response in KD [8]. These results highlight the involvement of dysregulated immune response in the pathogenesis of KD.

Genetic association of KD with inositol 1,4,5-trisphosphate 3-kinase C (ITPKC) in a genome wide scan has been recently reported by Onouchi et al. They first indicated a functional SNP in the *ITPKC* gene associated with KD susceptibility and the development of coronary artery lesions [9]. The polymorphism of *ITPKC* (rs28493229) located in intron 1 that resulted in the different transcriptional level of mature mRNA by interfering the RNA splicing efficiency. *ITPKC* is a kinase that phosphorylates inositol 1,4,5-trisphosphate (IP_3_). IP_3_ is an important factor in the initiating of calcium release from endoplasmic reticulum [10].

The empty of endoplasmic further evokes store-operated calcium influx. Store-operated calcium influx has been shown to involve in the activation of T cells. For example, IP_3_-mediated calcium signaling pathways can activate the translocation of nuclear factor of activated T-cells (NFAT) that further evoked the release of cytokines and the initiation of immune responses [10]. ITPKC is a negative regulator of Ca^2+^/NFAT signaling pathways. High level expression of ITPKC can attenuate the Ca^2+^/NFAT pathways via over phosphorylation of IP_3_ that resulted in reduction of T cell activation and IL-2 production. *ITPKC* (rs28493229) was confirmed to associate with the susceptibility of Kawasaki disease and CAL formation. However, the replication studies of *ITPKC* (rs28493229) from Taiwanese populations were controversial [11], [12].

In this study, we examined the association between the *ITPKC* (rs28493229) and the risk of KD using a case-control study. The relationship of clinical data including CAL, intravenous immunoglobulin (IVIG) treatment response, and aneurysm formation with genetic polymorphisms of *ITPKC* were also evaluated.

## Materials and Methods

### Patients studied

All subjects studied were children with the diagnosis of KD and were admitted at Chang Gung Memorial Hospital at Kaohsiung between 2001 and 2009. All patients were treated with a single infusion of IVIG (2 g/kg) administered over a 12-hour period. Aspirin (3–5 mg/kg/day) was administered until all signs of inflammation were resolved or regression of CAL was detected under two-dimensional (2D) echocardiography; these conditions were the same as that in our previous studies [6], [7], [8], [13], [14,]. We excluded patients who did not meet the diagnostic criteria for KD. CAL was defined by the internal diameter of the coronary artery being at least 3 mm (4 mm, if the subject was over the age of 5 years) or the internal diameter of a segment being at least 1.5 times that of an adjacent segment, as observed in the echocardiogram [15], [16], [17]. KD patients with coronary artery ectasia or dilatation which was disappearing within the initial 2 months after the onset of illness were defined as transient ectasia (or transient CAL) and not judged as CAL. IVIG responsiveness was defined as defervescence 48 h after the completion of IVIG treatment and no fever (temperature, >38°C) recurrence for at least 7 days after IVIG with marked improvement or normalization of inflammatory signs. A total of 1190 control subjects were recruited from the general population who volunteered to participate in our study while receiving a health screening examination at the Kaohsiung Medical University Hospital. All the subjects gave the consent form. The study protocol conformed to the Declaration of Helsinki and study was approved by the Institute Review Board of Hospital.

### DNA extraction

Blood cells were subjected to DNA extraction by treating them first with 0.5% SDS lysis buffer and then protease K (1 mg/ml) for digestion of nuclear protein for 4 h at 60°C. Total DNA was harvested by using the Gentra extraction kit followed by 70% alcohol precipitation.

### Genotyping

Genomic DNA was extracted from whole blood samples by using the standard method. Genotyping was carried out using the TaqMan Allelic Discrimination Assay (Applied Biosystems, Foster city, CA). Briefly, the polymerase chain reaction (PCR) was performed by using a 96-well microplate with the ABI 9700 Thermal Cycler. The thermal cycle conditions were as follows: denaturing at 95°C for 10 min, followed by 40 cycles of denaturing at 92°C for 15 s and annealing and extension at 60°C for 1 min. After PCR, fluorescence was measured and analyzed using the System SDS software version 1.2.3. Average genotyping successful rate in our lab is around 95.7%, so some individuals are without genotype data.

### Statistical analysis

SAS 9.1 for Windows was used for analysis. The statistical differences between cases and controls in genotype and allele frequency were assessed by the χ^2^-test or the Fisher exact test. The statistical differences in the genotype and allele frequency of KD patients with and those without CAL formation and patients responding to IVIG and those showing resistance were assessed using the χ^2^-test. For the meta-analysis, sufficient data were available from other two KD association studies, and the odds ratios with 95% confidence intervals were calculated for the risk factors investigated. Combined odds ratios were calculated using Mantel–Haenszel method with a fixed effect model for each outcome measure. Statistical analysis was completed using the rmeta package of R software.

## Results

### Meta-analysis shows that *ITPKC* SNP rs28493229 is associated with the susceptibility of KD

**Figure 1:**
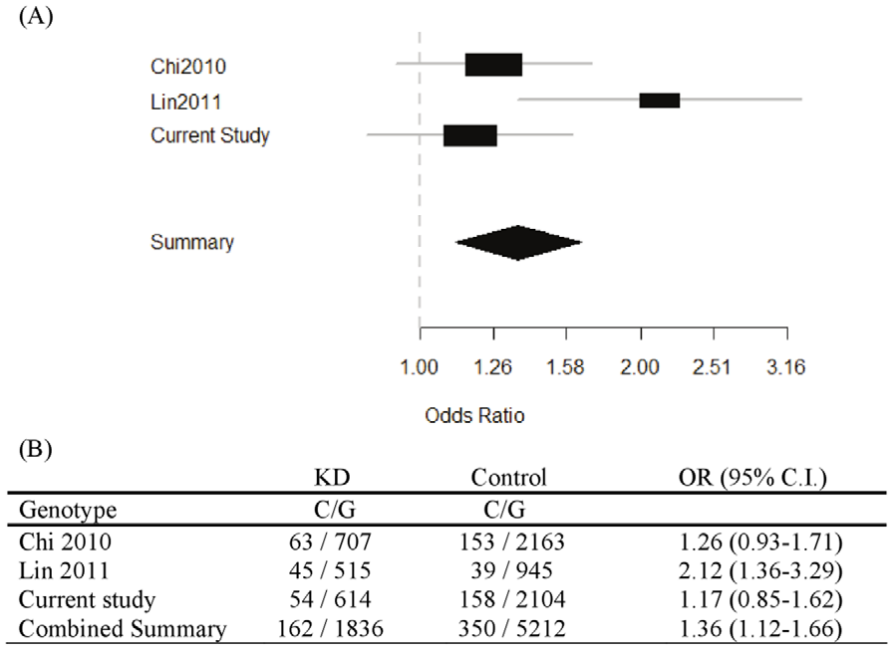
Forest Plots. (**A**) Depicting the association between KD patients and controls. (**B**) Allele frequency of rs28493229 in KD patients and controls in different cohortsfrom Taiwan.

Table 1 shows the characteristics of the subjects. A total of 341 KD patients and 1190 controls were recruited in this study. 66.3% of cases and 57.2% of controls were male. The mean age of patients and controls was 1.6±1.4 months (standard deviation [SD]) and 26.0±22.6 years, respectively. Children were predominant in the study population, an observation that is in agreement with the reported incidence of KD. The prevalence of KD is less than 1/1,000 children in Taiwan. Therefore, we assumed that there was no KD case in the control group. There were 10.3% of KD with CAL formation, and 4.1% with aneurysm formation. Among these KD patients 12.6% were resistant to initial IVIG treatment. The distribution of *ITPKC* SNP genotypes was in accordance with the Hardy-Weinberg equilibrium for both cases and controls (Table 2). However, the SNP tested in this study doesn't show a significant association with KD under three genetic models (Dominant model; Recessive model and Allelic model). To assess the role of variation in *ITPKC* SNP rs28493229 more extensively, we therefore performed a meta-analysis on all published KD association studies in the Taiwanese population including the present data. The results showed a significant association between *ITPKC* SNP rs28493229 and the susceptibility of KD (OR = 1.36, 95% CI 1.12–1.66) in the Taiwanese population (Figure 1).

**Table 1:** Basal characteristics of patients with Kawasaki disease (KD) and of normal controls.

Characteristics	Patients with KD	Normal Control
Number of subjects	341	1190
Gender: male, No (%)	226 (66.3%)	681 (57.2%)
Age (years)	1.6±1.4	26.0±22.6
Range	3.5-144 (months)	1–85 (years)
Coronary artery lesions (CAL)	35 (10.3%)	
Aneurysm formation	14 (4.1%)	
Intravenous immunoglobulin (IVIG) resistant	43 (12.6%)	

CAL: coronary artery lesions; IVIG: intravenous immunoglobulin; SD: standard deviation.

**Table 2:** Genotype frequencies for *ITPKC* SNP and Kawasaki disease susceptibility.

	Genotype	Case (%) (n = 334)	Control Subjects (%) (n = 1131)	Allele	Case (%) (n = 334)	Control Subjects (%) (n = 1131)	Genotype *P* Value	Dominant *P* Value	Recessive *P* Value	Allelic *P* Value
rs28493229	CC	2 (0.6)	8 (0.7)	C	54 (8.1)	158 (7.0)	0.509	0.283	0.832	0.335
	CG	50 (15.0)	142 (12.6)	G	614 (91.9)	2104 (93.0)		0.062†	0.891†	0.065†
	GG	282 (84.4)	981 (86.7)							

† Adjusted the effects of age and gender.

**Table 3:** Genotyping and allele frequency of *ITPKC* SNP in patients with Coronary artery lesion (CAL) and without CAL.

	Genotype	CAL (%) (n = 34)	Without (%) (n = 295)	Allele	CAL (%) (n = 34)	Without (%) (n = 295)	Genotype *P* Value	Dominant *P* Value	Recessive *P* Value	Allelic *P* Value
Rs28493229	CC	1 (2.9)	1 (0.3)	C	7 (10.3)	45 (7.6)	0.181	0.674	0.065	0.440
	CG	5 (14.7)	43 (14.6)	G	61 (89.7)	545 (92.4)				
	GG	28 (82.4)	251 (85.1)							


### No association between *ITPKC* SNP rs28493229 and CAL formation or IVIG treatment response in KD patients

A total of 341 KD patients were included in this study, of which 35 patients (10.3%) had CAL formation and 43 patients (12.6%) were resistant to initial IVIG treatment **(** Table 1 **)**. We further evaluate the relationship between rs28493229 and the risk of CAL formation or IVIG resistance. The data is shown in Table 3 and Table 4, respectively. The frequency of C allele was higher in the patients with CAL formation (10.3% vs. 7.6%) or IVIG resistance (11.6% vs. 7.6). However, the genotype or allele frequency of rs28493229 still failed to reach any significant association with CAL formation or IVIG resistance.

**Table 4:** Genotyping and allele frequency of *ITPKC* SNP in patients with resistant and responsive to Intravenous immunoglobulin (IVIG) treatment.

	Genotype	Resistant (%) (n = 43)	Responsive (%) (n = 291)	Allele	Resistant (%) (n = 43)	Responsive (%) (n = 291)	Genotype *P* Value	Dominant *P* Value	Recessive *P* Value	Allelic *P* Value
Rs28493229	CC	0 (0.0)	2 (0.7)	C	10 (11.6)	44 (7.6)	0.266	0.159	0.581	0.226
	CG	10 (23.3)	40 (13.7)	G	76 (88.4)	538 (92.4)				
	GG	33 (76.7)	249 (85.6)							

### SNP rs28493229 of *ITPKC* significant associated with aneurysm formation in KD

We further identify the role of SNP rs28493229 of *ITPKC* in the pathogenesis of CAL in KD patients. A subset analysis where cases reported having aneurysm formation was performed. In this study, 14 patients (4.1%) had aneurysm formation (aneurysm was defined as internal diameter >4 mm or in children ≧5 years of age, the internal diameter of a segment being at least 1.5 times that of an adjacent segment and that was persistent after at least one year followed up) (Table 1). As shown in the Table 5, SNP rs28493229 of *ITPKC* was associated with the KD patients with aneurysm formation. KD patients carry rs28493229 homozygous C/C genotype had a 24.5-fold (*P* = 0.001) increased risk of aneurysm formation when compared with those with C/G and G/G genotypes.

**Table 5:** Genotyping and allele frequency of *ITPKC* SNP in patients with aneurysm or without aneurysm.

	Genotype	Aneurysm (%) (n = 14)	Without (%) (n = 320)	Allele	Aneurysm (%) (n = 14)	Without (%) (n = 320)	Genotype *P* Value	Dominant *P* Value	Recessive*P* Value	Allelic *P* Value
Rs28493229	CC	1 (7.1)	1 (0.3)	C	3 (10.7)	51 (8.0)	**0.004** *	0.892	**0.001** *	0.602
	CG	1 (7.1)	49 (15.3)	G	25 (89.3)	589 (92.0)				
	GG	12 (85.8)	270 (84.4)							

*Significant (*P<0.05*) values are in bold.

## Discussion

Kawasaki disease is an acute vasculitis that may lead to acquired coronary artery aneurysms. Many genetic factors have been reported to involve in the development of coronary artery aneurysms. A major advance came when Onouchi et al. performed a large scale of genome wide screening to study the polymorphisms in KD patients [9]. They found that C allele of *ITPKC* (rs28493229) that reduced the expression of ITPKC associated with KD susceptibility and formation of coronary artery lesions from Japan and US cohort. Not only did it indicated that genetic factors contributed to the susceptibility as well as the pathogenesis of KD, but this was the first unequivocal identification of the functional SNP in a new gene (*ITPKC*). However, the replication studies of *ITPKC* (rs28493229) from Taiwanese populations were with controversial results. Chi et al. [11], didn't find any statistically significant association between the *ITPKC* SNP rs28493229 and KD susceptibility or CALs in Taiwanese children. Interestingly, reports from Lin et al. [12], indicated that C-allele of *ITPKC* SNP rs28493229 is associated with KD susceptibility and BCG scar reactivation during the acute phase but not associated with the develop or severity of CAL formation. As shown in Table 2, although rs28493229 didn't have significant association with the KD, higher C allele frequency in KD cases (8.1% vs 7.0%) was seen in this study. To increase the analysis power, we therefore performed a meta-analysis on all published KD association studies in the Taiwanese population. Consistent with the first ITPKC studies by Onouchi [11], [12], results from meta-analysis strongly indicated that C allele of *ITPKC* rs28493229 is a risk allele in the KD susceptibility in the Taiwanese population. In this study, we also first analysis the association between rs28493229 with IVIG treatment response and aneurysm formation. Our results indicated that C allele of *ITPKC* rs28493229 had significant higher risk to have aneurysm formation. Although the exact role of ITPKC in the development of KD is unclear, it may function as the negative regulator in the regulation of immune genes or to be involved in the T cell activation mechanisms. The results also, at least, reflect the facts that complex genetic, ethnic, and environmental factors are involved in the association with KD [11], [12]. Regarding to the different results obtained for *ITPKC* genetic association studies in the Taiwanese population, we attribute this to the population migration between Taiwan and China, Thailand, Indonesia, Malaysia, Vienna and other Asia areas, due to the increase of genetic diversity between cities in the south or north of Taiwan.

ITPKC is able to effect cellular expression level of IP_3_ via phosphorylation steps. Two possible signaling pathways that may involve in the increase of intracellular calcium concentration can be triggered by IP_3._ First, upon binding to the IP_3_ receptor (IP_3_R) on the endoplasmid reticulum, IP_3_ triggers the release of intracellular calcium from stores. Second, store empty resulted in the activation of STIM1-mediated store-operated calcium channels. Both calcium releases from stores or calcium influx through store-operated calcium channels have been confirmed to initiate signaling pathways to regulate inflammatory cytokines and immune diseases. In T-cells, agents block calcium influx through store-operated calcium channel attenuated the translocation of NFAT or NFkB and the expression of immune related genes [18]. The polymorphism of *ITPKC* (C allele) exhibited lower gene expression that may result in the increase of global intracellular calcium concentration via IP_3_-mediated pathways. Over-activation of calcium-dependent inflammatory cytokines may damage coronary vessel wall and cause to the aneurysm formation. Our results implied that C-allele of *ITPKC* SNP rs28493229 may passively increase the activation of calcium-dependent signaling pathways that lead to aneurysm formation in KD patients.

KD is considered as one of the immune-mediated diseases. Several genes have been reported to be associated with susceptibility to KD and/or CAL formation including *ITPKC*, *CASP3, CTLA4, TGF-beta* pathways and *MMP* family… etc [9], [16], [19], [20], [21], [22], [23]. Genes of *ITPKC* and *CASP3* are involved in the Ca^2+^/NFAT pathways. These two genes have been shown to associate with susceptibility to KD in the Japanese and European Americans. Consistent with this, we did observe a significant association in the KD patients with aneurysm formation even though the allele frequency is low. What might be the relevance of our findings to the KD? KD patients who are resistant with initial IVIG therapy have higher risk of developing CAL or aneurysm formation. Many inhibitors for Ca^2+^/NFAT pathways are already in the clinical use. From pharmacology prospective, to identify novel inhibitors against Ca^2+^/NFAT pathways seems to be promising in the treatment of KD. Further pharmacogenomic studies to confirm to the effects of inhibitors for Ca^2+^/NFAT pathways such as Cyclosporine A in the IVIG resistant patients may should be considered.

In conclusion, we reported that *ITPKC* (rs28493229) had significant association with the susceptibility of KD in the Taiwanese population. Importantly, KD patients carry *ITPKC* rs28493229 C allele had significant higher risk to have aneurysm formation.

## References

[1] Kawasaki T, Kosaki F, Okawa S, Shigematsu I, Yanagawa H (1974). A new infantile acute febrile mucocutaneous lymph node syndrome (MLNS) prevailing in Japan.. Pediatrics.

[2] Huang WC, Huang LM, Chang IS, Chang LY, Chiang BL (2009). Epidemiologic features of Kawasaki disease in Taiwan, 2003-2006.. Pediatrics.

[3] Wang CL, Wu YT, Liu CA, Kuo HC, Yang KD (2005). Kawasaki disease: infection, immunity and genetics.. Pediatr Infect Dis J.

[4] Newburger JW, Takahashi M, Gerber MA, Gewitz MH, Tani LY (2004). Diagnosis, treatment, and long-term management of Kawasaki disease: a statement for health professionals from the Committee on Rheumatic Fever, Endocarditis, and Kawasaki Disease, Council on Cardiovascular Disease in the Young, American Heart Association.. Pediatrics.

[5] Burns JC, Glode MP (2004). Kawasaki syndrome.. Lancet.

[6] Liang CD, Kuo HC, Yang KD, Wang CL, Ko SF (2009). Coronary artery fistula associated with Kawasaki disease.. Am Heart J.

[7] Kuo HC, Wang CL, Liang CD, Yu HR, Chen HH (2007). Persistent monocytosis after intravenous immunoglobulin therapy correlated with the development of coronary artery lesions in patients with Kawasaki disease.. J Microbiol Immunol Infect.

[8] Kuo HC, Wang CL, Liang CD, Yu HR, Huang CF (2009). Association of lower eosinophil-related T helper 2 (Th2) cytokines with coronary artery lesions in Kawasaki disease.. Pediatr Allergy Immunol.

[9] Kuo HC, Yang KD, Liang CD, Bong CN, Yu HR (2007). The relationship of eosinophilia to intravenous immunoglobulin treatment failure in Kawasaki disease.. Pediatr Allergy Immunol.

[10] Onouchi Y, Gunji T, Burns JC, Shimizu C, Newburger JW (2008). ITPKC functional polymorphism associated with Kawasaki disease susceptibility and formation of coronary artery aneurysms.. Nat Genet.

[11] Putney JW (2009). Capacitative calcium entry: from concept to molecules.. Immunol Rev.

[12] Chi H, Huang FY, Chen MR, Chiu NC, Lee HC (2010). ITPKC gene SNP rs28493229 and Kawasaki disease in Taiwanese children.. Hum Mol Genet.

[13] Lin MT, Wang JK, Yeh JI, Sun LC, Chen PL (2010). Clinical Implication of the C Allele of the ITPKC Gene SNP rs28493229 in Kawasaki Disease: Association With Disease Susceptibility and BCG Scar Reactivation..

[14] Kuo HC, Liang CD, Wang CL, Yu HR, Hwang KP (2010). Serum albumin level predicts initial intravenous immunoglobulin treatment failure in Kawasaki disease.. Acta Paediatr.

[15] Kuo HC, Wang CL, Wang L, Yu HR, Yang KD (2008). Patient characteristics and intravenous immunoglobulin product may affect eosinophils in Kawasaki disease.. Pediatr Allergy Immunol.

[16] Akagi T, Rose V, Benson LN, Newman A, Freedom RM (1992). Outcome of coronary artery aneurysms after Kawasaki disease.. J Pediatr.

[17] Shimizu C, Matsubara T, Onouchi Y, Jain S, Sun S (2010). Matrix metalloproteinase haplotypes associated with coronary artery aneurysm formation in patients with Kawasaki disease.. J Hum Genet.

[18] Shulman ST, De Inocencio J, Hirsch R (1995). Kawasaki disease.. Pediatr Clin North Am.

[19] Ishikawa J, Ohga K, Yoshino T, Takezawa R, Ichikawa A (2003). A pyrazole derivative, YM-58483, potently inhibits store-operated sustained Ca2+ influx and IL-2 production in T lymphocytes.. J Immunol.

[20] Kuo HC, Yu HR, Wang CL, Lin IC, Liu CA (2010). CTLA-4, position 49 A/G polymorphism associated with coronary artery lesions in Kawasaki disease.. J Clin Immunol.

[21] Kuo HC, Yu HR, Juo SH, Yang KD, Wang YS (2010). CASP3 gene single nucleotide polymorphism (rs72689236) and Kawasaki disease in Taiwanese children.. Journal of Human Genetics.

[22] Onouchi Y, Ozaki K, Buns JC, Shimizu C, Hamada H (2010). Common variants in CASP3 confer susceptibility to Kawasaki disease.. Hum Mol Genet.

[23] Shimizu C, Jain S, Lin KO, Molkara D, Frazer JR (2010). Transforming Growth Factor-{beta} Signaling Pathway in Patients with Kawasaki Disease.. Circ Cardiovasc Genet.

